# Training for a (half‐)marathon: Training volume and longest endurance run related to performance and running injuries

**DOI:** 10.1111/sms.13725

**Published:** 2020-06-03

**Authors:** Tryntsje Fokkema, Ankie A.D.N. van Damme, Maarten W.J. Fornerod, Robert‐Jan de Vos, Sita M.A. Bierma‐Zeinstra, Marienke van Middelkoop

**Affiliations:** ^1^ Department of General Practice Erasmus MC University Medical Center Rotterdam The Netherlands; ^2^ Department of Cell Biology Erasmus MC University Medical Center Rotterdam The Netherlands; ^3^ Department of Orthopeadics and Sports Medicine Erasmus MC University Medical Center Rotterdam The Netherlands

**Keywords:** athletic performance, physical endurance, prevention and control, prospective study

## Abstract

**Objective:**

Examine the associations of training volume and longest endurance run with (half‐)marathon performance and running‐related injuries (RRIs) in recreational runners.

**Materials and Methods:**

During the preparation for and directly after the running event, 556 participants of a half marathon and 441 participants of a marathon completed three questionnaires on RRIs, average weekly training volume and the longest endurance run. With finish time, decline in pace during the running event and RRIs as dependent variables, linear and logistic regression analyses were performed to test the associations with weekly training volume and the longest endurance run.

**Results:**

In half‐marathon runners, a high training volume (>32 km/wk) (β −4.19, 95% CI: −6.52;−1.85) and a long endurance run (>21 km) (β −3.87, 95% CI: −6.31;‐−1.44) were associated with a faster finish time, while a high training volume (β −1.81, 95% CI: −3.49;−0.12) and a long endurance run (β −1.89, 95% CI: −3.65;−0.12) were also related to less decline in pace. In marathon runners, a low training volume (<40 km/wk) was related to a slower finish time (β 6.33, 95% CI: 0.18;12.48) and a high training volume (>65 km/wk) to a faster finish time (β −14.09, 95% CI: −22.47;−5.72), while a longest endurance run of <25 km was associated with a slower finish time (β 13.44, 95% CI: 5.34;21.55). No associations between training characteristics and RRIs were identified.

**Conclusions:**

Preparation for a (half‐)marathon with a relatively high training volume and long endurance runs associates with a faster finish time, but does not seem related to an increased injury risk.

## 1 INTRODUCTION

Over the last few decades, long‐distance running grew in popularity, with more athletes participating in running events like marathons and half marathons.^1^, ^2^ For example, 15 450 athletes ran the Dutch Rotterdam Marathon in 2017, compared with only 200 in 1981. Traditionally, training for a (half‐)marathon involves a high training volume and long endurance runs. This way of training seems beneficial for (half‐)marathon performance, since a high training volume is, together with a high training pace, related to a better marathon performance time.^3^, ^4^ However, a high training volume is also associated with a higher risk of running‐related injuries (RRIs).^5^ Running more than 65 km/wk for men and between 48 and 63 km for women was found to be related to a higher risk of RRIs in recreational runners.^5^ It has therefore been suggested that injuries may be prevented by reducing the training volume.^6^, ^7^

For runners and their trainers, it is a challenge to find a training volume that is high enough for an optimal (half‐)marathon performance, but not that high it will increase the risk on injuries. Currently, there is a trend in the Netherlands that runners train for a marathon with a high training intensity and training runs of maximal 14 km. It is claimed that this way of training decreases the injury risk, but has no effect on finish time. There are some indications that replacing a small percentage of the endurance training sessions with high interval training improves endurance performance.^8^ However, the effects of replacing all long endurance runs with training runs of high intensity and low volume remain unknown. More scientific knowledge on the associations between training, performance, and RRIs may give insight if this type of preparation is indeed as successful as claimed. So far most studies aimed to investigate the association between training and performance or between training and RRIs. To our best knowledge, performance and injury risk are not yet investigated together in one study. Therefore, the aim of this study was to examine the associations of training volume and longest endurance run with (half‐)marathon performance and RRIs in recreational runners participating in a half marathon or marathon.

## 2 MATERIALS AND METHODS

The present study was part of the INSPIRE trial, a randomized‐controlled trial on the effectiveness of an online injury prevention program.^9^, ^10^ Because the injury prevention program had no effect on the number of RRIs, this study can be interpreted as a cohort.^10^ The INSPIRE trial was funded by the Netherlands Organization for Health Research and Development (ZonMW, 536001001) and was performed in collaboration with Golazo Sports, an organization of large running events in the Netherlands. The trial was approved by the Medical Ethical Committee of the Erasmus MC University Medical Center Rotterdam, the Netherlands (MEC‐2016‐292).

Potential participants of this study were runners who registered for the half marathon of the NN City Pier City Run The Hague or the NN Marathon Rotterdam in 2017. On the online registration form for these running events, runners were informed about the study and were asked to indicate if they were interested in participating in the trial. If runners registered for both the half marathon and marathon, only their first registration was taken into account. Runners who were interested in participating and met the inclusion criteria (aged 18 years and older and registration at least 2 months before the running event) received additional information about the study and were asked to give digital informed consent and subsequently complete the online baseline questionnaire. Two weeks before, 1 day after and 1 month after the running event follow‐up questionnaires were sent to the participants by e‐mail.

In the baseline, questionnaire information on demographics (age, sex, weight, and height) and training characteristics (running experience [years], being member of an athletics association (yes/no) and the type of training (percentage endurance training, interval training, and exercises) and sustaining an RRI in the 12 months before baseline (yes/no)] was collected. In all three follow‐up questionnaires, participants were asked to indicate if they sustained a new RRI since completing the previous questionnaire (yes/no) and if yes, the location of the RRI was recorded. An RRI was defined as an injury of the muscles, joints, tendons, and/or bones in the lower back or lower extremities (hip, groin, thigh, knee, leg, ankle, foot, and toes) that was caused by running. Furthermore, one of the following criteria had to be met: (a) the injury was severe enough to cause a reduction in running distance, speed, duration, or frequency for at least 1 week, (b) the injury led to a visit of a doctor and/or physiotherapist, and/or (c) medication was necessary to reduce symptoms as a result of the injury. The first follow‐up questionnaire (2 weeks before the running event) also covered average training characteristics over the last month. These training characteristics included average weekly training volume (km), frequency (times per week), and duration (minutes). Furthermore, information on the longest endurance run before the running event (km) and average training pace (minutes per km) was collected.

Body mass index (BMI) was calculated using weight and height. Weekly training volume and longest endurance run were categorized following the existing literature.^11^, ^12^, ^13^ When literature was lacking, averages were used as cutoff points. Consequently, for marathon runners, weekly training volume was categorized into <40, 40‐65, and >65 km and the longest endurance run into <25, 25‐30, 30‐35, and >35 km. For half‐marathon runners, weekly training volume was categorized into <20, 20‐32, and >32 km and the longest endurance run into <15, 15‐21, and >21 km. Performance times of the participants (finish time and interval times of every 5 km) were provided by the organization of the running events. The decline in pace during the running event was defined as the percentage difference in interval time from 5‐10 and 15‐20 km for half‐marathon runners and the percentage difference in interval time from 5‐10 to 35‐40 km the marathon runners.

Only runners who completed both the baseline and the first follow‐up questionnaire were included in the analyses. Differences in baseline characteristics between participants who did and did not complete the first follow‐up questionnaire and between included half‐marathon and marathon runners were tested using independent *t* test, Mann‐Whitney *U* tests, and chi‐square tests. For the analyses involving finish time and decline in pace during the running event, only runners who finished the running event were included. For the analyses of the RRIs, also runners that did not start and/or finish the running event were included.

Descriptive statistics (frequencies and percentages for categorical data; mean and standard deviation [SD] or median and interquartile range [IQR] for numeric data) were calculated for all collected data. Differences in characteristics of the participants within the weekly training volume, longest endurance, and average training pace groups were determined with univariate linear and logistic regression analyses. To visualize the relationship between training characteristics and finish time and decline in pace, scatterplots were created. To determine the associations between these variables, two separate multivariable linear regression analyses were performed with the training characteristics as independent variables and finish time and decline in pace, respectively, as dependent variable. The associations between the training characteristics and new RRIs during follow‐up were determined using multivariable logistics regression analysis with the training characteristics as independent variables and a new RRI during follow‐up as dependent variable. All regression analyses were adjusted for possible confounders including sex, age, BMI, running experience, and RRI in 12 months before baseline. The analyses were performed separately for the half‐marathon and marathon runners in SPSS Statistics 24. *P*‐values below .05 were regarded as statistically significant.

## 3 RESULTS

**FIGURE 1:**
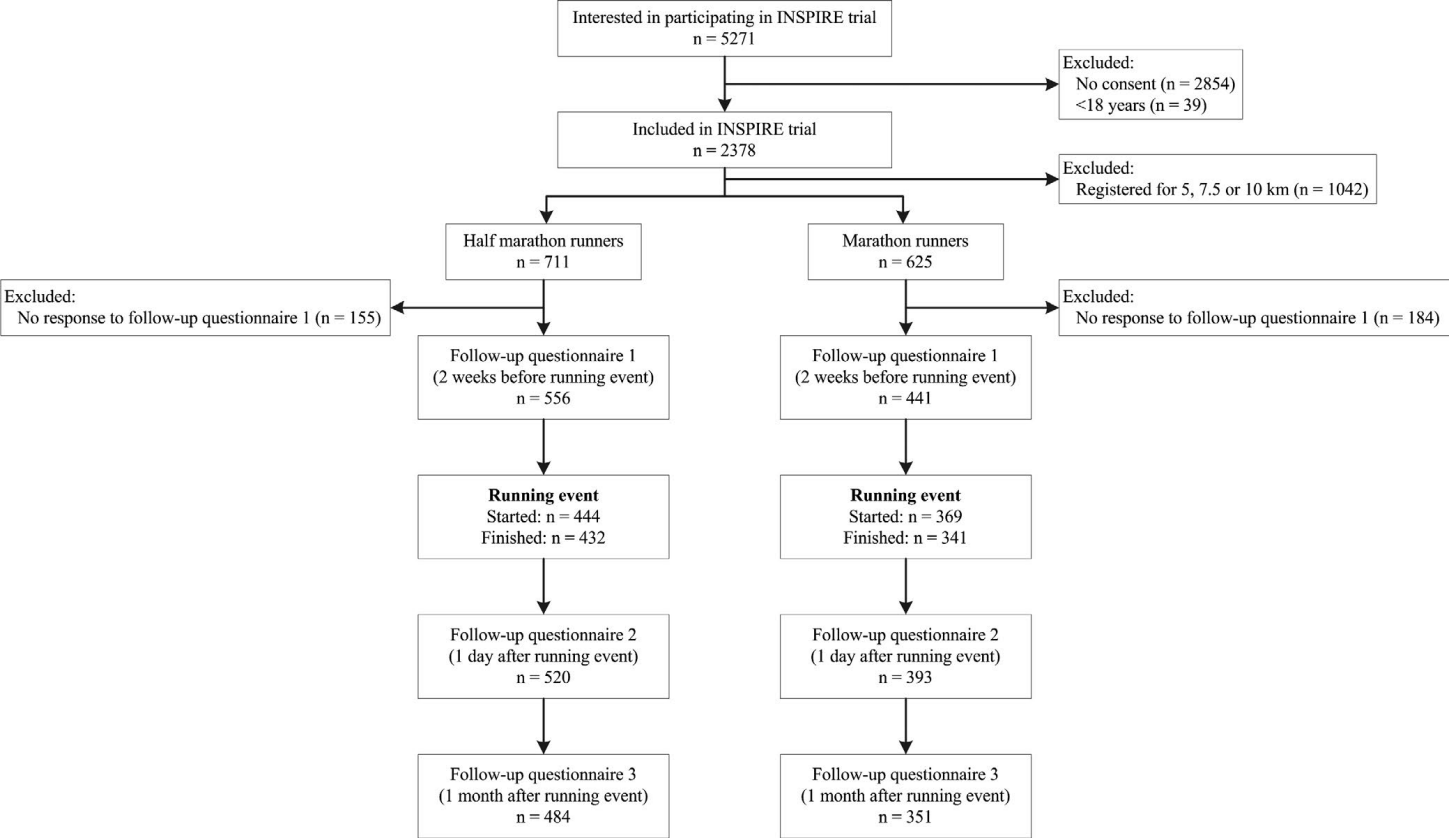
Flowchart of the participants

A total of 1336 half‐marathon and marathon runners participated in the INSPIRE trial and completed the baseline questionnaire (Figure 1). Of these long‐distance runners, 339 participants (25.4%) did not fill out the first follow‐up questionnaire and were therefore excluded from the analyses of the current study. The runners that were included in the analyses were on average older (42.2 [SD 11.7] vs 39.5 [SD 10.7] years, *P* < .01), had a lower BMI (23.1 [SD 2.4] vs 23.6 [SD 2.6] kg/m^2^, *P* < .01), longer experience with running (7.8 [SD 8.3] vs 6.8 [SD 7.3] years, *P* = .02), and were more often member of an athletic association (36.5% vs 27.7%, *P* < .01) than the runners that were excluded from the analyses.

The 997 runners included in the analyses were on average 42.2 (SD 11.7) years old, and the majority (65%) was male (Table 1). In total, 556 half‐marathon runners were included in the analyses. They ran on average 29.9 (SD 19.4) km/wk, with a training pace of 5:45 (SD 0:45) minutes per km and a longest endurance run of 19.3 (SD 6.5) km, and finished their race on average in 2:00:05 (SD 0:16:41) hours, with an average decline of 11.1% (SD 7.4). A total of 268 half‐marathon runners sustained an RRI (48.2%), of which most were located in the knee (13.5%) (Appendix 1). The 441 included marathon runners had an average weekly training volume of 43.6 (SD 27.3) km, a longest endurance run of 29.1 (SD 8.5) km, and a training pace of 5:41 (SD 0:44) minutes per km. They finished their race in 4:17:54 (SD 0:37:14) hours with a decline of 25.0% (SD 15.4). In total, 243 (55.1%) marathon runners sustained a new RRI during follow‐up. Most RRIs were located in the knee (17.2%). The characteristics of the participants divided by the training characteristics are presented in Table 2.

**TABLE 1:** Characteristics of the participants (frequencies (%)/average (SD)/median [IQR])

	Half‐marathon runners	Marathon runners
N	556 (55.8%)	441 (44.2%)
Demographic characteristics		
Sex (male)	339 (61.0%)	309 (70.1%)^a^
Age (years)	42.8 (12.1)	41.4 (11.1)
BMI (kg/m^2^)	23.1 (2.3)	23.1 (2.5)
Training characteristics^b^		
Running experience (years)	5.0 [2.5;10.0]	5.0 [3.0;9.2]
Weekly training distance (km)	26.0 [20.0;40.0]	40.0 [30.0;50.0]^a^
Weekly training frequency	3.0 [2.0;3.0]	3.0 [3.0;4.0]^a^
Training pace (minutes per km)	5:45 [5:20;6:00]	5:40 [5:15;6:00]
Longest endurance run before running event (km)	18.5 [15.5;22.0]	32.0 [27.0;35.0]^a^
Type of training (%)		
Endurance training	70.3 (21.5)	67.2 (20.5)^a^
Interval training	22.3 (18.4)	25.3 (17.3)^a^
Exercises	6.8 (8.9)	7.5 (8.8)^a^
Member of athletic association (yes)	191 (34.4%)	173 (39.2%)
Injuries		
RRI in 12 mo before baseline (yes)	291 (52.3%)	241 (54.6%)
Running event		
Started running event (yes)	444 (79.9%)	369 (83.7%)
Finished running event (yes)	432 (77.7%)	341 (77.3%)
Finish time (hours)	2:00:05 (0:16:41)	4:17:54 (0:37:14)^a^
Decline during running event (%)	11.1 (7.4)	25.0 (15.4)^a^

^a^ Significant different (*P* < .05) from half‐marathon runners.

^b^ 2‐6 wk before the running event.

**TABLE 2:** Characteristics of the participants divided by the training characteristics (frequencies (%)/average (SD)/median [IQR])

Half marathon	Weekly training volume (km)^d^			Longest endurance run (km)			Training pace (min/km)^e^		
	< 20	20‐32 (reference)	> 32	< 15	15‐21 (reference)	> 21	< 5:15	5:15‐6:00 (reference)	> 6:00
N	129	233	193	94	310	152	120	294	134
Sex (female)	61 (47.3%)	89 (38.2%)	67 (34.7%)	42 (44.6%)	135 (43.5%)	40 (26.3%)^a^	13 (10.8%)^a^	119 (40.5%)	83 (61.9%)^a^
Age (years)	38.7 (11.3)^a^	43.3 (12.0)	42.8 (12.1)	42.4 (11.6)	42.9 (12.6)	42.8 (11.4)	39.5 (11.0)^a^	42.9 (12.0)	45.5 (12.6)^a^
BMI (kg/m^2^)	23.4 (2.3)	23.1 (2.3)	23.0 (2.3)	23.5 (2.2)	23.3 (2.3)	22.6 (2.3)^a^	22.1 (1.6)^a^	23.0 (2.2)	24.3 (2.5)^a^
Running experience (years)	5.0 [2.0;8.0]	5.0 [2.7;10.0]	5.0 [3.0;10.0]	5.0 [2.0;10.0]	4.4 [2.5;10.0]	5.6 [3.0;10.0]	5.0 [2.5;12.5]	5.0 [3.0;10.0]	4.3 [2.2;9.0]
RRI in 12 mo before baseline (yes)	67 (51.9%)	124 (53.2%)	99 (51.3%)	51 (54.3%)	165 (53.2%)	75 (49.3%)	69 (57.5%)	150 (51.0%)	65 (48.5%)
Finish time (hours)^b^	2:05:32 (0:15:12)	2:02:03 (0:16:24)	1:55:26 (0:16:29)^a^	2:06:48 (0:17:28)	2:03:28 (0:15:35)	1:51:31 (0:15:16)^a^	1:41:31 (0:09:29)^a^	2:00:12 (0:11:55)	2:17:47 (0:11:23)^a^
Decline in pace during event (%)^b^, ^c^	12.1 (8.7)	12.0 (7.4)	9.7 (6.7)^a^	10.3 (9.7)	12.1 (7.1)	9.4 (6.9)^a^	11.4 (7.6)	11.0 (6.9)	11.3 (8.3)
Running‐related injury during follow‐up (yes)	74 (57.3%)	109 (46.8%)	84 (43.5%)	55 (58.5%)	152 (49.0%)	61 (40.1%)	46 (38.3%)	143 (48.6%)	74 (55.2%)

Marathon	Weekly training volume (km)^d^			Longest endurance run (km)				Training pace (min/km)^f^		
	< 40	40‐65 (reference)	> 65	< 25	25‐30	30‐35 (reference)	> 35	< 5:15	5:15‐6:00 (reference)	> 6:00
N	158	239	43	91	111	200	38	103	224	97
Sex (female)	48 (30.4%)	78 (32.6%)	6 (14.0%)^a^	28 (30.8%)	30 (27.0%)	68 (34.0%)	6 (15.8%)^a^	10 (9.7%)^a^	66 (29.5%)	52 (53.6%)^a^
Age (years)	39.7 (11.4)^a^	42.0 (11.1)	44.3 (9.9)	41.5 (11.0)	39.9 (12.1)	41.7 (10.7)	43.5 (10.7)	37.7 (10.0)^a^	41.9 (11.1)	44.4 (10.9)^a^
BMI (kg/m^2^)	23.4 (2.6)	23.1 (2.4)	22.5 (2.8)	23.5 (2.8)	23.2 (2.5)	23.0 (2.4)	22.5 (2.4)	22.1 (2.1)^a^	23.1 (2.3)	24.1 (3.0)^a^
RRI in 12 mo before baseline (yes)	83 (52.5%)	136 (56.9%)	22 (51.2%)	62 (68.1%)^a^	57 (51.4%)	105 (52.5%)	16 (42.1%)	49 (47.6%)	131 (58.5%)	49 (50.5%)
Running experience (years)	4.0 [2.0;8.0]^a^	5.0 [3.1;10.0]	5.8 [2.5;10.0]	4.2 [1.8;8.0]	4.0 [3.0;10.0]	5.0 [3.0;9.8]	5.4 [3.0;10.0]	5.0 [3.0;10.0]	5.0 [3.0;10.0]	4.2 [2.0;6.8]
Finish time (hours) ^b^	4:31:03 (0:36:28)^a^	4:19:04 (0:33:20)	3:43:03 (0:35:43)^a^	4:37:43 (0:35:02)^a^	4:25:07 (0:39:14)^a^	4:15:26 (0:34:34)	3:50:18 (0:29:13)^a^	3:38:45 (0:22:55)^a^	4:21:05 (0:26:24)	4:58:59 (0:23:50)^a^
Decline in pace during event (%)^b^, ^c^	29.1 (15.9)^a^	23.9 (15.4)	21.9 (12.5)	28.8 (14.8)	26.2 (16.5)	24.5 (14.2)	20.1 (17.9)	24.0 (15.9)	25.4 (15.6)	24.6 (14.0)
Running‐related injury during follow‐up (yes)	98 (62.0%)	127 (53.1%)	18 (41.9%)	59 (64.8%)	66 (59.5%)	98 (49.0%)	19 (50.0%)	58 (56.3%)	117 (52.2%)	56 (57.7%)

^a^ Significant different (*P* < .05) from reference group.

^b^ Runners who did not finish the running event were removed from this analysis.

^c^ Decline in pace missing for one participant.

^d^ Weekly training volume missing for one participant.

^e^ Training pace missing for 8 participants.

^f^ Training pace missing for 17 participants.

The scatterplots of the training characteristics and finish time and decline in pace are shown in Appendix 2. The multivariable analyses showed that in half‐marathon runners, a training volume of more than 32 km/wk, a longest endurance run of more than 21 km, and a training pace of <5:15 min/km 2‐6 weeks before the running event were associated with a faster finish time, while a training pace of more than 6:00 min/km was associated with a slower finish time (Table 3). Furthermore, a training volume of more than 32 km/wk and a longest endurance run of more than 21 km were associated with less decline in pace during the race. In marathon runners, a training volume of <40 km/wk, a longest endurance run of <25 km, and a training pace of more than 6:00 min/km were associated with a slower finish time, while a training volume of more than 65 km/wk and training pace of <5:15 min/km were associated with a faster finish time. No significant associations between training characteristics and decline in pace were found in marathon runners. In both half‐marathon and marathon runners, none of the training variables were associated with new RRIs.

**TABLE 3:** Results of the multivariable regression analyses on the associations of the training characteristics with (half‐)marathon performance and running‐related injuries

	Finish time (minutes)^a^		Decline in pace during event (%)^a^		Running‐related injury	
	Β	95% CI	β	95% CI	OR	95% CI
Half marathon						
Sex (female)	**7.63** **	**5.05;10.21**	−0.78	−2.64;1.09	0.99	0.62;1.59
Age (years)	**0.22** **	**0.11;0.32**	**0.10**	**0.02;0.17**	1.00	0.98;1.02
BMI (kg/m^2^)	**1.54** **	**1.03;2.05**	**0.39**	**0.03;0.76**	1.02	0.94;1.12
Running experience (years)	−0.07	−0.21;0.06	−0.06	−0.16;0.04	1.01	0.98;1.03
RRI in 12 mo before baseline (yes)	1.38	−3.31;0.54	−0.35	−1.73;1.03	**2.12** **	**1.49;2.99**
Weekly training volume (km)						
<20	1.87	−0.96;4.70	0.90	−1.14;2.93	1.41	0.86;2.32
20‐32	Reference		Reference		Reference	
>32	−**4.19** **	−**6.52;** −**1.85**	−**1.81** *	−**3.49;** −**0.12**	0.97	0.63;1.50
Longest endurance run (km)						
<15	0.09	−3.39;3.58	−2.31	−4.81;0.20	1.19	0.69;2.04
15‐21	Reference		Reference		Reference	
>21	−**3.87** **	−**6.31;** −**1.44**	−**1.89** *	−**3.65;** −**0.12**	0.83	0.52;1.30
Training pace (min/km)						
<5:15	−**13.63** **	−**16.34;** −**10.93**	1.22	−0.723.17	0.68	0.41;1.11
5:15‐6:00	Reference		Reference		Reference	
>6:00	**12.51** **	**9.89;15.12**	−0.59	−2.48;1.30	1.23	0.77;1.98
Marathon						
Sex (female)	**10.78** **	**4.16;17.40**	−**7.05** *	−**11.46;0.31**	1.00	0.59;1.70
Age (years)	0.25	−0.02;0.53	−0.01	−0.19;0.18	1.00	0.98;1.02
BMI (kg/m^2^)	**1.58** *	**0.38;2.79**	−0.33	−1.14;0.47	1.04	0.95;1.14
Running experience (years)	−**0.42** *	−**0.77;** −**0.06**	−0.21	−0.44;0.02	0.99	0.97;1.02
RRI in 12 mo before baseline (yes)	−1.62	−6.70;3.46	0.91	−2.44;4.27	**2.59** **	**1.71;3.91**
Weekly training volume (km)						
<40	**6.33** *	**0.18;12.48**	3.66	−0.42;7.75	1.29	0.79;2.09
40‐65	Reference		Reference		Reference	
>65	−**14.09** **	−**22.47;** −**5.72**	−1.75	−7.21;3.71	0.58	0.28;1.19
Longest endurance run (km)						
<25	**13.44** **	**5.34;21.55**	1.68	−3.66;7.02	1.00	0.53;1.89
25‐30	6.40	−0.04;12.82	0.21	−4.04;4.55	0.75	0.45;1.25
30‐35	Reference		Reference		Reference	
>35	−4.86	−13.51;3.79	−4.23	−9.88;1.41	1.01	0.44;2.32
Training pace (min/km)						
<5:15	−**33.67** **	−**40.40;** −**26.93**	−2.61	−7.02;1.80	1.56	0.90;2.71
5:15‐6:00	Reference		Reference		Reference	
>6:00	**30.47** **	**23.52;37.42**	−0.15	−4.79;4.49	1.26	0.73;2.18

Bold indicates statistical significant association (*P* < .05).

^a^ Runners who did not finish the running event were removed from these analyses.

* *P* < .05.

** *P* < .01.

## 4 DISCUSSION

The aim of this study was to examine the associations of training volume and longest endurance run with (half‐)marathon performance and RRIs in recreational runners. The results showed that in half‐marathon runners, a higher training volume, longer longest endurance run, and higher training pace were related to a faster finish time, while a higher training volume and longer longest endurance run were also related to less decline during the race. These parameters were not associated with the onset of RRIs. In marathon runners, a lower weekly training volume, shorter longest endurance run, and slower training pace were associated with a slower finish time, while a higher weekly training volume and faster training pace were related to a faster finish time. Also in marathon runners, no associations between training characteristics and RRIs were found.

Previous research on (half‐)marathon performance focused primarily on the prediction of finish time based on a variety of demographic, physiological, and training characteristics. Of the training characteristics, mean weekly training volume and training pace were strongly related to finish time.^3^, ^4^, ^14^ The present study confirms these findings. One may expect that faster runners also tend to run with higher training volumes, which may affect the relation between training volume and finish time. However, the multivariable linear regression analysis also included training pace and a high weekly training volume was still strongly associated with finish time. Furthermore, additional analyses revealed only weak correlations between mean weekly training volume and training pace (half marathon: *r* = −.171; marathon: *r* = −.201). These findings indicate that finish time is determined by a combination of training volume and training pace. A high weekly training volume and a fast training pace seem both beneficial for finish time. Also the length of the longest endurance run was associated with finish time, where longer endurance runs seem beneficial for finish time. However, a longest endurance run of more than 35 km was not associated with better performance compared with a longest endurance run of 30‐35 km. For a fast marathon finish time, it therefore seems important to train with a high weekly training volume, but it does not seem necessary to include an endurance run of more than 35 km.

In addition to (half‐)marathon finish time, decline in pace during the event was also examined as a performance outcome. This was suggested as a proxy variable for running fatigue, since a positive association exists between decline in pace and muscle breakdown markers.^15^ Marathon runners have more decline in pace during the event, which was confirmed in the current study.^16^, ^17^ Furthermore, Haney et al showed that slower marathon finishers had more decline in pace than faster marathon finishers.^18^ This seems to suggest the relation between a high training volume, and a fast finish time is due to less decline in pace. The results of the current study contradict this suggestion. In both half‐marathon and marathon runners, only weak correlations existed between decline in pace and finish time (half marathon: *r* = .208; marathon: *r* = .294). Furthermore, in the marathon runners there was a significant association between training volume and finish time, but not between training volume and decline in pace. However, in the half‐marathon runners, a relation between training volume and decline in pace was found. Therefore, the results of this study indicate that decline in pace during a running event does not seem to be a good performance outcome measure in marathon runners.

In this study, no associations between the training characteristics and RRIs were found. This finding contradicts with some previous studies, in which a high training volume was related to a higher injury risk.^5^, ^11^ This may be partly explained by the relatively low number of marathon runners in the highest training volume and longest endurance run groups (n = 43 and n = 38, respectively). However, also in the half‐marathon runners no associations between training characteristics and RRIs were identified, while these runners were more equally divided in training volume and longest endurance run. Furthermore, there have also been some other studies that found no associations between training volume and injury risk or a high training volume was even protective for RRIs.^19^, ^20^ These conflicting findings indicate that the relation between training volume is complex and may be confounded by other factors. It has been suggested that “survival of the fittest” may be an important confounder of the relation between training volume and RRIs.^19^ Possibly only runners who are least prone for RRIs prepare for a (half‐)marathon with a high training volume and long endurance runs, while runners who are prone to RRIs may be forced to reduce their training volume due to beginning RRIs. However, additional analyses of our data showed no significant associations between training volume and previous RRIs in the 12 months before the INSPIRE trial. Therefore, it cannot be confirmed that “survival of the fittest” is a confounder for the relation between training volume and RRIs in the current study. Furthermore, Bertelsen et al^21^ suggested that the development of RRIs depends on the relation between the structure‐specific load capacity and structure‐specific cumulative load per training session. Because the structure‐specific load capacity adapts to the applied training load, the progression in training volume may play an important role in the development of RRIs.^5^, ^22^ Therefore, future research on the complex relation between training volume and RRIs should also take the progression in training volume into account. As suggested by Nielsen et al^23^, time‐to‐event models could be used when analyzing these data, since these methods are well suited to deal with changes in training load as a time‐varying exposure.

A strength of the current study is that it is the first study that investigated the relations of both (half‐)marathon performance and RRIs with training characteristics. Furthermore, this study included a large sample of both half‐marathon and marathon runners. However, some limitations should be taken into account when interpreting the results of this study. First, 82 (14.7%) of the runners included as half‐marathon runners also participated in the marathon and were therefore actually preparing for a marathon. This is a potential source of bias, because significant differences between half‐marathon and marathon runners existed in baseline and training characteristics. Performing the analyses without the half‐marathon runners that participated in both events showed similar results as the analyses with these runners for finish time and RRIs. For decline in pace, the results were slightly different: There was no significant association with training volume anymore when analyzing the data without the runners that participated in both running events. Another limitation is the relative high number of runners that were excluded from the analyses. The excluded runners were younger, had a higher BMI and less running experience than the included runners. In previous literature, all these factors were associated with an increased injury risk.^24^, ^25^ Furthermore, a higher BMI was associated with a slower half‐marathon time.^26^, ^27^ Therefore, results of the current study may be biased by excluding runners from the analyses. Also the use of self‐reported measures is a limitation. However, because nowadays approximately 75% of the runners track their training sessions with GPS, runners may be quite accurate in reporting their training characteristics.^28^ Another possible limitation of this study is that the identified associations of training pace, training volume, and the longest endurance run with finish time are possibly confounded by the intrinsic speed (“talent”) of runners. Also, the efficacy of novel training schedules with lower training volume and higher training intensities cannot be assessed from these data, because of the limited contribution of these training methods in the sample. Future research would benefit from including intrinsic training intensity (eg, heart rate) as a variable.

## 5 PERSPECTIVE

Results of this study indicate that a high weekly training volume, long endurance runs, and a fast training pace are beneficial for both half‐marathon and marathon performance. For half‐marathon runners, an endurance run of more than 21 km may have a positive effect on finish time. For a fast marathon finish time, a high training volume of at least 40 km/wk seems important. However, it does not seem necessary to include an endurance run of more than 35 km. In both half‐marathon and marathon runners, training volume and the distance of the longest endurance run were not related to injury risk.

## CONFLICT OF INTEREST

The authors declare to have no conflicts of interest.

## APPENDIX 1

Number of injured runners per anatomical injury location for all participants (n = 997) and the half‐marathon runners (n = 556) and marathon runners (n = 441) separately

**Table:**

	All participants		Half‐marathon runners		Marathon runners	
	N	%	N	%	N	%
New injury during follow‐up (yes)	511	51.3	268	48.2	243	55.1
Number of new injuries	797		416		381	
Anatomical injury location						
Lower back	50	5.0	27	4.9	23	5.2
Buttock	50	5.0	23	4.1	27	6.1
Hip	59	5.9	30	5.4	29	6.6
Groin	39	3.9	19	3.4	20	4.5
Ventral thigh	22	2.2	10	1.8	12	2.7
Dorsal thigh	53	5.3	35	6.3	18	4.1
Knee	151	15.1	75	13.5	76	17.2
Shin	40	4.0	15	2.7	25	5.7
Calf	104	10.4	57	10.3	47	10.7
Achilles tendon	60	6.0	27	4.9	33	7.5
Ankle	65	6.5	35	6.3	30	6.8
Foot	86	8.6	53	9.5	33	7.5
Toe	18	1.8	10	1.8	8	1.8

## APPENDIX 2

Scatterplots of the actual data

**Half‐Marathon Runners**

**Marathon Runners**
